# Systematic Review of Behaviour Change Theories Implementation in Dietary Interventions for People Who Have Survived Cancer

**DOI:** 10.3390/nu13020612

**Published:** 2021-02-13

**Authors:** Jana Sremanakova, Anne Marie Sowerbutts, Chris Todd, Richard Cooke, Sorrel Burden

**Affiliations:** 1 School of Health Sciences, University of Manchester, Oxford Road, Manchester M13 9PL, UK; annemarie.sowerbutts@manchester.ac.uk (A.M.S.); chris.todd@manchester.ac.uk (C.T.); Sorrel.Burden@manchester.ac.uk (S.B.); 2 Academic Health Science Centre, Manchester M13 9PL, UK; 3 Manchester University NHS Foundation Trust, Manchester M13 9WL, UK; 4 NIHR Applied Research Collaboration Greater Manchester, Manchester M13 9NQ, UK; 5 Department of Psychology, University of Liverpool, Liverpool L69 3B, UK; R.Cooke4@liverpool.ac.uk; 6 Salford Royal NHS Foundation Trust, Manchester M6 8HD, UK

**Keywords:** cancer, survivorship, diet, anthropometry, behaviour change, BCT(s)

## Abstract

Background: An increasing number of dietary interventions for cancer survivors have been based on the behaviour change theory framework. The purpose of this study is to review the use and implementation of behaviour change theories in dietary interventions for people after cancer and assess their effects on the reported outcomes. Methods: The search strategy from a Cochrane review on dietary interventions for cancer survivors was expanded to incorporate an additional criterion on the use of behaviour change theory and updated to September 2020. Randomised controlled trials (RCT) testing a dietary intervention compared to the control were included. Standard Cochrane methodological procedures were used. Results: Nineteen RCTs, with 6261 participants (age range 44.6 to 73.1 years), were included in the review. The Social Cognitive Theory was the most frequently used theory (15 studies, 79%). Studies included between 4 to 17 behaviour change techniques. Due to limited information on the mediators of intervention and large heterogeneity between studies, no meta-analyses was conducted to assess which theoretical components of the interventions are effective. Conclusions: Whilst researchers have incorporated behaviour change theories into dietary interventions for cancer survivors, due to inconsistencies in design, evaluation and reporting, the effect of theories on survivors’ outcomes remains unclear.

## 1. Introduction

The term cancer survivor defines a person living with and beyond cancer [1]. In this article, we define cancer survivors as people who completed all the active treatments and are in the recovery period. Adherence to a healthy lifestyle, including eating a healthy diet and being physically active, has been associated with a reduction in overall mortality among cancer survivors [2,3]. It has been suggested that the experience of cancer diagnosis and cancer treatment stimulates survivors’ motivation to change their lifestyle [4]. This is supported by qualitative studies indicating that people are willing to make changes to their lifestyle and have different needs and preferences for support after treatment [5,6,7,8,9]. Hence, identification of the most effective approaches to help people who experienced cancer change their lifestyle is crucial for achieving improvement in their health outcomes.

There is a growing interest in utilising behaviour change theories to inform the content and design of interventions. A large body of scientific evidence suggests that interventions addressing change in health behaviours are more likely to achieve success if they are developed with a clear understanding of the targeted behaviour, its environmental context, and if the intervention incorporates a theoretical basis [10,11,12,13,14].

A number of systematic reviews have identified the most frequently used theories in health interventions [15,16,17]. These reviews show that the selection of theories has not changed over the last 20 years, with the most frequently used theories being Social Cognitive Theory (SCT) [18], the Trans Theoretical Model (TTM; also known as the Stages of Change) [19], and the Health Belief Model (HBM) [20].

The extent to which theories are implemented within interventions varies. An intervention can be classified as either informed by theory, applying theory, testing theory, building or creating theory [14]. In addition, theories can be applied at different levels targeting individuals, groups, organisations or communities [21].

A number of concerns have been raised around the validity and reliability of theories used to address health behaviour, their application, interpretation, and translation into applied research and clinical practice. In summary, a number of researchers [22,23,24,25,26] have drawn attention to ‘’how we use theory, how we test theory, how we translate theories into interventions, and what conclusions we draw from research’’ [14] and ask for clarification and transparency of behaviour change interventions [24].

To understand how theoretical components work in an intervention, there is a need for authors to provide a detailed report of how the theory has been used to inform the intervention. Moreover, there should be an identification of the components of the intervention using a taxonomy of behaviour change techniques (BCTs) [11,22,27], such as the one reported by Michie et al. (2013) [28]. This can help with the understanding of behaviour change tools used in the interventions. Although, BCTs are not directly linked with a specific theory, they can have implications for theory when there is an obvious link between the theory and BCTs.

When theory is applied to interventions, it is important to conduct both process evaluation, alongside the usual outcome evaluation. The outcome evaluation tests the efficacy of an intervention, by looking for positive changes in measures of physiological (body fat), health (survival), and behavioural outcomes (fruit intake). Although sometimes studies depend on surrogate outcomes, such as the intention to change behaviour, which are mediators or determinants of behaviour. The process evaluation identifies how the intervention is designed to work and how changes in the determinants of behaviour (e.g., self-efficacy) [24] mediate the effect of the intervention on outcomes.

In cancer survivorship research, encouraging results have been demonstrated in systematic reviews of interventions that have utilised the behaviour change theory (ies) to increase exercise [22] and improve the quality of life [29]. In dietary interventions for cancer survivors, only one study assessed using dietary changes as outcomes for SCT based interventions and indicated promising improvement in dietary behaviour [27]. Since then, theories have been widely used to inform the design and development of behaviour change interventions [30]. To date, however, little is known about the degree of use and implementation of behaviour change theories and BCTs in dietary interventions for cancer survivors. Hence, the aims of this review are as follows: (1) To identify what theories have been used; (2) to establish the extent of implementation of behaviour change theories and BCTs; (3) to identify what behaviour change outcomes are reported; and (4) to report on the effects of theories and BCTs on outcomes in dietary interventions for people after cancer.

## 2. Materials and Methods

This systematic review was registered with PROSPERO, number CRD42020172444 and follows the standard of Cochrane systematic reviews [31] and the Preferred Reporting Items for Systematic Reviews and Meta-analysis (PRISMA) [32].

### 2.1. Literature Searching

**Figure 1:**
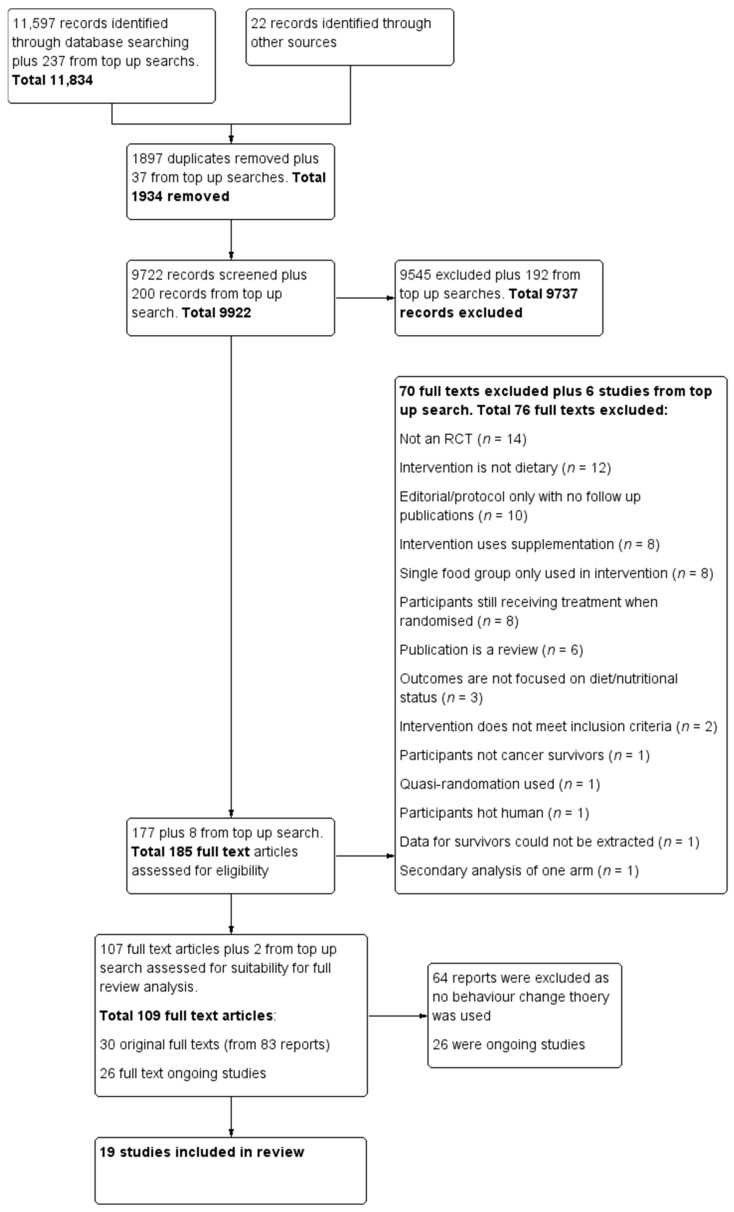
Preferred reporting items for systematic reviews and meta-analysis (PRISMA) flow diagram for the study selection.

This systematic review extends the information collected for the Cochrane review on dietary interventions for cancer survivors [30]. During the completion of the Cochrane review, the question on implementation of behaviour change theory emerged. However, behaviour change was not within the scope of the review and agreed protocol. Searches conducted for a previous Cochrane review were updated on 30 September 2020 and an additional inclusion criterion on the use of behaviour change theory was incorporated. We have included only RCTs, however, 11 trials are final reports of large projects [33,34,35,36,37,38,39,40,41,42,43] that included non-randomised studies and qualitative work. The following databases were searched: The Cochrane Central Register of Controlled trials; Medline via Ovid; Embase via Ovid; the Allied and Complementary Medicine Database; the Cumulative Index to Nursing and Allied Health Literature; and the Database of Abstracts of Reviews of Effects. We also searched other resources, other reviews on the topic, and the International Trials Registry for ongoing trials. Search words were matched with the MESH term truncation systems and Boolean operators “and” with “or” function were used. The search strategy is attached as Supplementary Table S1. The results of the literature searches were uploaded to Covidence, (https://www.covidence.org/ (accessed on 10 December 2020), Melbourne, AU) an online software for data management. Data were checked for duplicates by the software and then manually. The titles and abstracts were independently screened by both Jana Sremanakova and Sorrel Burden based on inclusion and exclusion criteria. Full texts were obtained to identify the eligible publications and checked by both Jana Sremanakova and Anne Marie Sowerbutts Any discrepancy during the process of screening was discussed between Jana Sremanakova, Sorrel Burden, and Anne Marie Sowerbutts. The search results and selection process have been recorded in the PRISMA (Preferred Reporting Items for Systematic Reviews and Meta-Analyses) flow diagram (Figure 1).

### 2.2. Study Inclusion Criteria

The primary criterion for this review was the use of behaviour change theory in the intervention design. Participants were adult (age > 18) survivors of cancer who completed surgery and all anticancer treatments. Only randomised control trials (RCT) testing a dietary intervention compared to a control group following standard care were included. The dietary intervention was defined as an oral nutritional intervention based only on promoting a healthy diet. Interventions were excluded if they were based on a single food group, oral supplements, including those with single or multiple nutrients and probiotic supplements, as well as studies using intravenous nutrient solutions including both enteral and parenteral nutrition. Dietary interventions using any delivery method: Group sessions, telephone instruction, written materials, mobile application, or web-based approaches were included.

### 2.3. Outcomes

The main outcomes of interest were the dietary behaviours and surrogate outcomes. Dietary behaviour included changes in energy consumption, nutrients, food groups identified by using food frequency questionnaires, dietary recall, food diaries or assessed by dietary assessment methods, including changes in anthropometry and body composition. Mediators of behaviour (surrogate outcomes) included psychological constructs (questionnaire-based score assessing constructs such as self-efficacy, intention or similar), readiness to change and goal settings (number of goals set).

### 2.4. Data Extraction and Risk of Bias Assessment

**Figure 2:**
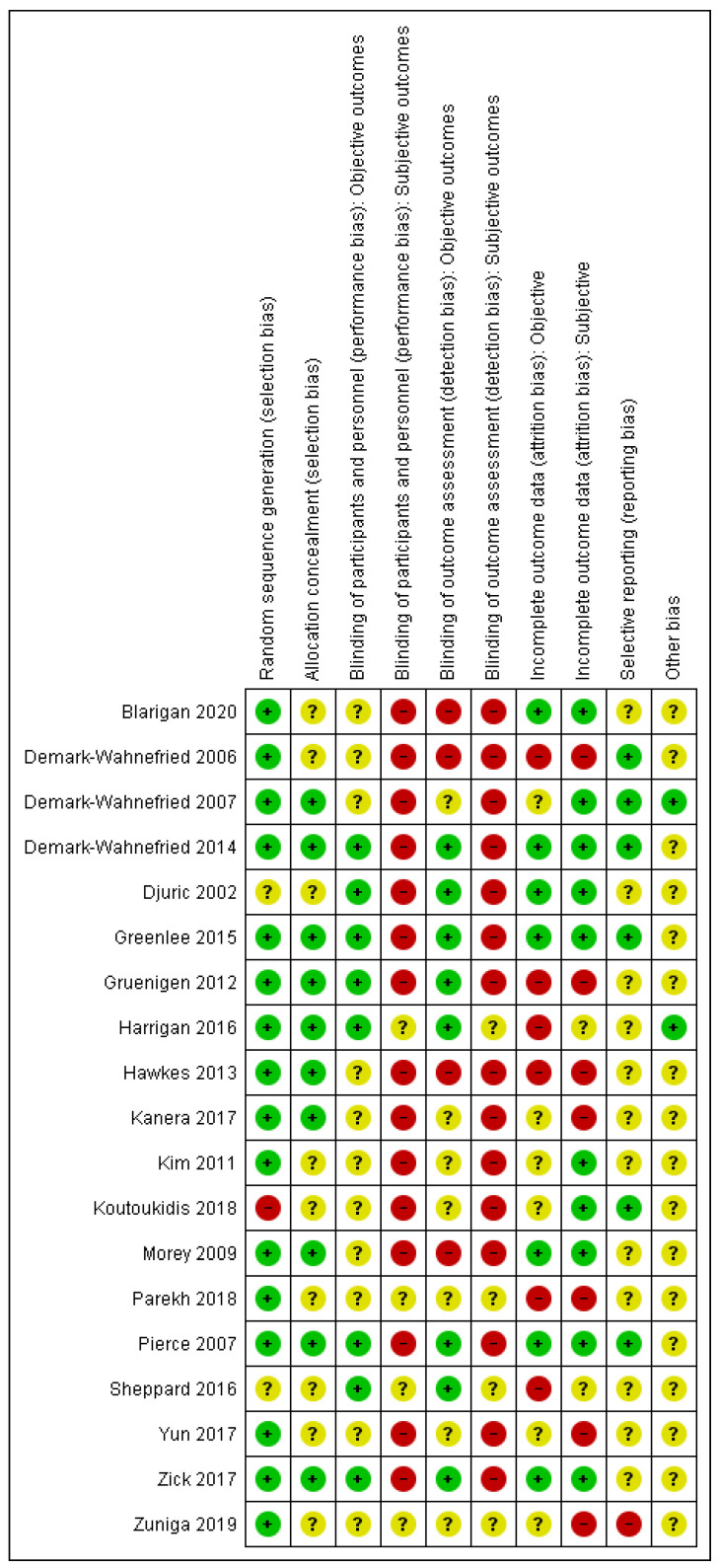
Risk of bias summary for each included study. Note: green (+)—low risk of bias, red (-)—high risk of bias, yellow (?)—unclear risk of bias.

A data extraction form was devised based on a Cochrane template [30]. One author (Jana Sremanakova) extracted data on study characteristics (author, publication year, full title, location, funding, study design, and duration), population characteristics (age, gender, type of treatment, cancer site, and stage), cancer site, dietary intervention provided, behaviour change theory, level of implementation of behaviour change approach in design, intervention resources, targeted behaviour or constructs, measured behaviour or constructs, form of testing behaviour change approach, outcomes of behavioural change, and adherence measures. Twenty percent of data extraction was double checked by all the co-authors. The BCT taxonomy [28] was used to identify the BCTs in the interventions. Jana Sremanakova independently coded BCTs for all the publications. In addition, all the co-authors coded 25% of publications and all the authors discussed with JS a final selection of BCTs. The risk of bias was assessed using the Cochrane Collaboration’s risk of bias tool [44]. The quality of evidence was generally low to very low, however, some outcomes were assessed as being of a moderate-certainty of evidence using GRADE [45] (See Supplementary Tables S2 and S3 for the GRADE assessment, and Figure 2 for the assessed risk of bias).

### 2.5. Statistical Methods

We used standard Cochrane methodological procedures and completed the meta-analysis in the Cochrane Review manger version 5.4.1 software (UK) [46]. The mean difference (MD) and 95% confidence intervals (CI) were calculated using a random effect statistical model. Heterogeneity of any combined studies was assessed by using I² (heterogeneity). If I² was greater than 30%, we examined possible reasons for heterogeneity in relation to study participants and similarity of clinical parameters in studies. Data not suitable for the meta-analysis were reported narratively.

## 3. Results

### 3.1. Search Results

Nineteen studies met the inclusion criteria and were included in this review with two studies identified based on the updated search.

### 3.2. Studies Characteristics

A total of 6261 participants were included in studies on dietary interventions for cancer survivors which utilised behaviour change theory (ies). The mean age of participants reported was between 44.6 to 73.1 years old. The majority of studies recruited women with breast cancer, so the proportion of male (982, 16%) and female (5279, 84%) participants was unequal. Ten studies (53%) included participants after breast cancer and two studies combined breast and prostate cancer. Two studies focused on gynaecological cancer, two on colon cancer, and three studies included a mixed population. Fourteen studies were conducted in the USA, two studies in South Korea, one study in Australia, one in the United Kingdom, and one in the Netherlands. Details on participant characteristics are reported in Table 1.

Most studies used a combination of strategies including mailed intervention, telephone calls, group sessions, individual sessions, automated messages, web-based interventions, and newsletters. However, there was always a predominant strategy: Eight studies used group interventions [36,37,40,43,50,51,52,54], four studies used mailed interventions [33,34,35,41], three studies used telephone counselling [39,42,49], two studies used individual sessions [38,53], and two studies were a web-based intervention [47,48]. One study [33] considered the ethnic difference in the study population by tailoring the resources based on age, race, and style of coping with cancer such as cognitive avoider, helpless or hopeless. There was one study that focused on black ethnic survivors of breast cancer [51], one study on the Korean population [52], and one study developed the evidence-based programme targeting Hispanic breast cancer survivors [37].

Although the included studies were all dietary interventions, most (74%, 14 studies) also targeted changes in physical activity. The length of interventions ranged from 1.8 to 12 months of follow up, but one study had 7.3 years of follow up [42]. The dropout rate was 11% (692 out of 6407 participants randomised).

Seven studies used an attention control group and provided participants with untailored information booklets [33,34,35,37,53], newsletters, and cooking classes [42] or suggested participants to follow a weight loss programme on their own [36]. Six studies used a waiting list control group and provided participants with general information [41,47,49,50,51,52]. Six studies used the usual care group with no additional support [38,39,40,43,48,54].

Only eight studies (42%) published a protocol with a detailed description of the intervention [33,34,37,39,40,41,42,54]. Four studies (21%) did not publish a protocol but included a description of the intervention in the main paper [36,48,49,51]. Additionally, seven studies (37%) provided limited information on the intervention [35,38,43,47,50,52,53].

### 3.3. Theoretical Framework

#### 3.3.1. Implementation of Behaviour Change Theory in the Interventions

All 19 studies specified at least one theoretical framework. Social Cognitive Theory (SCT) was the most frequently used theory (15 studies, 79%), followed by the Trans-Theoretical Model of Change (TTM; nine studies, 47%), then the Theory of Planned Behaviour (TPB, three studies, 16%). One study reported using the Acceptance Commitment Model (ACM) and another reported using the Control Theory (CT). Ten studies (53%) based their intervention on two theories, most frequently SCT and the TTM. Eight studies (42%) used one theory, while one study used three theories (SCT, TPB, TTM). In addition to these theories, the Motivational Interviewing Technique (MIT) was used in three studies (three studies, 16%) [39,51,54]. The studies targeted changes in one or several constructs as part of the intervention. The most frequently targeted constructs were self-efficacy to gain confidence in the participants’ ability to change their lifestyle, goal settings targeting ability to select achievable goals, and behavioral capacity focusing on knowledge of a healthy lifestyle. Details of the studies’ characteristics are included in Table 2.

#### 3.3.2. Use and Reporting of Behaviour Change Techniques in the Interventions

The quality of reported details on how the intervention was performed varied between studies, and influenced our ability to map the BCTs used in the interventions. Based on the BCT taxonomy classification, interventions focused on the following BCTs: 9.1. Credible source (19 studies), 4.1. Instruction on how to perform the behaviour (18 studies), 3.1. Social support (17 studies), 1.3. Goal setting (12 studies), 2.3. Self-monitoring of behaviour (12 studies), 1.2. Problem solving (10 studies), and 2.2. Feedback on behaviour (10 studies). The studies used between four to 17 BCTs (see Table 3).

### 3.4. Dietary Behaviour Outcomes

Most studies assessed behaviour change by measuring differences in the dietary intake at the baseline and follow up. The most frequently reported dietary outcomes were changes in portions of fruit and vegetables, fibre and energy intake. Only seven studies provided the data suitable for meta-analysis [33,34,35,37,42,43,54]. Certainty of evidence is detailed in Supplementary Tables S2 and S3, and the analyses are presented in Supplementary Figures S1–S8.

For the dietary intake, we found no difference in energy, fruit, vegetable, fruit and vegetables, and fibre intake between the intervention group and control at 6 and 12 months. For adherence, three studies reported a Diet Quality Index. The analysis showed that dietary interventions compared to the control is likely to improve the Diet Quality Index (mean difference 3.62, 95% CI 1.95 to 5.30; three studies; 719 participants; moderate-certainty evidence) at 12 months. See Supplementary Figure S6—Analysis 1.6. Two studies were excluded from the analysis. One study reported the quality of the diet using a different index [40] and one study used a different scoring system [49].

For anthropometric measures, we found no difference in the body weight and waist circumference, but at 12 months, the dietary intervention versus control probably led to a slight decrease in the body mass index (mean difference −0.79 kg/m2, 95% CI −1.50 to −0.07; four studies; 777 participants; moderate-certainty evidence). Supplementary Figure S8—Analysis 2.2.

### 3.5. Mediators of Behaviour

Although several constructs were identified and used in the interventions, most included studies did not measure the mediators related to the theory. Therefore, it was not possible to determine if changes in outcomes were due to changes in the mediator variables. The included studies were designed to understand how an intervention changes behaviour, but not what aspect of the theory is positively related to the behaviour change and reported outcomes (i.e., the RCTs tended to be pragmatic rather than explanatory trials).

Only four studies attempted to address the mediators of behaviour [33,34,35,49]. In paper-based interventions with automated messages, self-efficacy for eating more fruit and vegetables was assessed at the baseline [33]. The study showed that more than 60% of the participants in both groups were already in very sure to an extremely sure stage of eating more fruit and vegetables at the start of the intervention.

One study using a tailored workbook with targeted telephone counselling reported self-efficacy for healthy eating [34]. This study showed that at 12 months, the number of people in the intervention group who were not sure at all about healthy eating decreased from 4.5% to 2.6%, those who were a little sure decreased from 2.3% to 1.3%, those who were somewhat sure decreased from 24.7% to 22.1%, those who were very sure increased from 43.8% to 57.1%, and those extremely sure decreased from 24.7% to 16.9%. Similar numbers were reported for the attrition control group who received a general workbook and general health recommendations through telephone counselling. One study [35] reported self-efficacy for adhering to a healthy weight loss diet as a score (very unsure = 5 to very sure = 1). At 12 months compared to the baseline, the score for the intervention group changed from 1.9 (standard deviation (SD) 0.8) to 2.1 (SD 0.9), while in the control group the score changed from 2.1 (SD 0.9) to 2.3 (SD 0.8). To provide formal tests of process evaluation, such results need analysing using inferential statistics.

The readiness to change defined based on the TTM as a precontemplation, contemplation, action, and maintenance stage was assessed in three studies. One study on a paper-based intervention with automated messages assessed the readiness to improve fruit and vegetable intake at the baseline and showed that around 60% of the participants were already in the preparation stage for change and around 30% in the action stage in both groups [33]. Two paper-based interventions with telephone support assessed the readiness to change the diet. One study [49] reported a difference between the baseline and three months and showed that in the intervention group, the number of people in the precontemplation stage and contemplation change decreased from 28% to 0, and from 50% to 11% in the preparation stage, while the number of people increased from 22% to 89% in the action or maintenance stage. In the control group, there was a decrease in the number of people in the pre-contemplation and contemplation stage from 28% to 22%, increase in the number of people in the preparation stage from 50% to 61%, and decrease in the number of people in the action or maintenance stage from 22% to 17%.

In the second study [34] at 12 months, the number of people in the intervention group in the pre-contemplative stage increased from 10.1% to 19.5%, in the contemplative stage decreased from 84.3% to 72.7%, and in the preparation stage the number increased from 5.6% to 7.8%. In the control group, there was an increase in people being in the pre-contemplative stage from 9.7% to 16.9%, 79% remained in the contemplative stage, and the number of people in the preparation stage decreased from 10.7% to 3.6%.

The paper-based interventions with tailored automated messages also evaluated the number of practiced goals at 12 months. The study reported that in the intervention group, the number of people with no practicing goal decreased from 116 to 59, and the number of people practicing two goals increased from 0 to 70, and the number of three goals from 0 to 15. The control group who received only general materials showed that the number of people with no practicing goal decreased from 115 to 90, the number of people practicing two goals increased from 0 to 41, and the number of people practising three goals from 0 to 7 [33].

## 4. Discussion

This systematic review established the use and implementation of behaviour change theories in dietary interventions for cancer survivors. SCT and TTM were the most frequently used theories, similar to other reviews of health behaviour change [12,22]. While all the included studies used theory to inform their intervention, due to the failure of most studies to measure constructs from such theories, it was impossible to determine how or if changes in targeted constructs, such as self-efficacy or readiness to change, mediated the effects of interventions on the reported outcomes. Therefore, only the meta-analyses of behavioural outcomes were conducted.

These analyses were based on telephone, group, and mailed interventions that used SCT, TTM or a combination of both. All the interventions also included the following BCTs—instructions on how to perform the behaviour, credibility of the source, and social support. Moreover, these meta-analyses are mostly applicable to the female population after breast cancer due to the prevalence of breast cancer studies.

The meta-analyses showed little or no difference in dietary outcomes, body weight, and waist circumference, as well as small changes in body mass index and likely an improvement in the diet quality index. A previous systematic review of dietary and physical activity interventions for cancer survivors applying SCT to the interventions concluded that SCT-based interventions provide promising results. However, no meta-analysis was conducted [27]. Weak changes in the dietary behaviour in studies using SCT and TTM in this review are not dissimilar from findings in the Cochrane review, which included also studies that did not incorporate any behaviour change theory [30].

Uncertain evidence and inconsistent findings may be related to many limitations in the interventions that remain unaddressed. Criticism of the conduct of behavioural interventions and suggestions for the best practice were reported some time ago [24,25,26,55]. However, many interventions aimed at people after cancer still do not adhere to these standards.

### 4.1. Intervention Design

It has been demonstrated that interventions addressing ethical, cultural, and environmental requirements in the targeted population are more effective [14]. In our review, one study considered the ethical differences in the study population and appropriately tailored resources [33] and one study developed an evidence-based programme targeting specifically Hispanic breast cancer survivors [37]. To design the tailored intervention for a specific population requires public involvement, piloting, and feasibility testing, which are often lacking in interventions. The possible reasons for the limited developmental work are funding and time constraints of the research. However, it has been recognised that piloting and feasibility testing is an important step to enhance the development of complex interventions, and Medical Research Council (MRC) guidance exists on the development and evaluation of complex interventions in healthcare [56]. In this review, only few interventions on breast cancer [33,37,42] and endometrial cancer [40] have been based on extensive developmental work before conducting the intervention.

### 4.2. Description of the Intervention

Currently, there is no standard way to report how theory is incorporated into dietary interventions and what behavioural aspects are addressed. Interventions claim to use theory, but fail to describe that theory in sufficient detail, and thus do not add to the knowledge accumulation and informed development of future studies. In essence, studies are pragmatic attempts to change behaviour, rather than explanatory trials attempting to understand the underlying theoretical mechanisms. For instance, it has been previously shown, that only 44% of 34 RCTs stated a theoretical basis for the intervention development [57] and only 50% of protocol-specified behaviour change techniques were reported in studies from Cochrane reviews on smoking cessation [58]. In our review, 58% of the studies did not publish a protocol, and 37% poorly reported on the intervention design, behaviour change theory implementation, and BCTs.

Similar to our review, others have also attempted to map BCTs used in intervention studies based on descriptions provided by the study authors, so as to report on the BCT use as accurately as possible [22,59]. An extended version of the CONSORT statement for reporting randomised trials of social and psychological interventions [60] exists. However, it omits to include details on how to report on the behaviour change theory implemented in the interventions and to address components based on BCT taxonomy. Hence, having consistent guidance for interventions on how and what to report about the theory incorporated in an intervention in the protocol and publication can bring clarity as well as improve the transparency of interventions [24].

### 4.3. Outcomes Reporting

The standardisation of outcomes reporting would allow for more robust pooling of data in the meta-analyses. In this review, studies reported data at different time points and presented data in different ways. Therefore, only seven studies out of 19 were included in the meta-analyses. If more studies would report at 3, 6, and 12 months intervals and provided more detailed descriptive data, it would improve the opportunity to conduct data synthesis and an effective comparison. For instance, exercise interventions for cancer survivors are conducted and reported reasonably consistently, and thus the meta-analyses with large numbers of studies have been conducted [22]. Researchers have been able to demonstrate that interventions were effective in achieving a modest increase in physical activity at 3 months. Furthermore, developing a core set of outcomes as ‘’an agreed, standardised and minimum set of outcomes that should be measured and reported in all clinical trials for a specific health condition’’ could improve the conduct of the meta-analyses [61] and thus provide fuller insight into the efficacy and effectiveness of interventions.

### 4.4. Assessment of the Behaviour Change Theory in Interventions

Although most interventions used theory, the majority of interventions did not assess the mediators of behaviour change related to theory (e.g., readiness to change) nor theory constructs (e.g., self-efficacy). Our review highlights that research teams are primarily focused on outcome evaluation and do not routinely conduct (or at least report) process evaluations to check that their interventions are working as per the design.

Only four studies out of 19 reported measures of such mediators. However, inconsistency in measurement meant it was not possible to meta-analyse these studies. The readiness to change and self-efficacy are important mediators that should be reported at the baseline. Grimmett et al. (2019) highlighted that if people enrolled in interventions are highly motivated to change their behaviour, a type two error occurs in the outcomes measurement irrespective of the group allocation, and small differences between the groups observed can mask the true effect of the intervention [22]. This has been demonstrated in the included studies which reported on self-efficacy and readiness to change at the baseline. These studies indicated that most participants are in the preparation or action stage or are fairly confident to make a change at the start of the intervention, and where groups are compared over time, small differences are observed.

Rejeski et al. (2000) also suggested that studies should report a readiness to change over time to provide the indication of success of the intervention [62]. Participants’ attitudes are likely to change during the intervention based on how they perceive the intervention to be difficult, and how they cope with it, and their level of motivation. This indicates that unless the mediators of behaviour such as self-efficacy and readiness to change are reported over time, the study’s outcomes can be misleading.

In summary, it remains unclear what theory works best or what constructs meaningfully influence the participants’ behaviour in dietary interventions for cancer survivors. Limitations such as lack of details on the applied theory, BCTs, and process evaluation in studies presented in this review prevent us from drawing any conclusion on the effectiveness of incorporating specific theories in interventions. Hence, better consistency in the design, evaluation, and reporting of dietary interventions for cancer survivors is required, in order to demonstrate not only how the implementation of theory supports the interventions, but also determine what is a cumulative effect of interventions on the targeted outcomes.

### 4.5. Limitations

Due to the limited consistency in reporting, all 19 authors would have had to be contacted to provide the details lacking on the behaviour change theory and BCT techniques used. We were unable to contact all the authors and hence, the review is based on publicly available information from studies and study protocols and thus highlights gaps in the quality of study reporting.

## 5. Conclusions

Currently, uncertainty exists on the impact of behaviour change theories implemented in dietary interventions for cancer survivors. There is a need for interdisciplinary work in the design, assessment, and monitoring of interventions in order to allow for the appropriate selection of theories, incorporation of the theory in the intervention design, as well as adequate reporting and testing of the theory to build up valid and consistent evidence. Active collaboration with a psychologist during the planning process and conduct of the trials may help in mitigating a number of issues discussed in this review and improve the efficacy of interventions.

## Figures and Tables

**Table 1:** Participant characteristics.

Authors	*n*	Mean Age (SD) (Years)	Gender (F:M ratio)	Ethnicity (%)	Higher education (%)	Cancer Site
Blarigan 2020 [47]	50	5.5 (3.5)	33/17	White 70%, Other 30%	College graduate 96%	Colorectal
Demark- Wahnefried 2006 [34]	182	71.5 (4.4) vs. 71.9 (5.6)	104/78	White 82.0% vs. 82.8% African American 14.6% vs. 15.0%, Other 3.4% vs. 2.2%	Not reported	Breast, prostate
Demark- Wahnefried 2007 [33]	543	57 (10.8)	304/239	White 83%, Black 13%, Other 4%	College graduate/ post graduate 58%	Breast, prostate
Demark- Wahnefried 2014 [35]	43	61.3 (7.4) **	43/0	Non-Hispanic White 74% Hispanic white 7%, African American 18%, Asian 1%	College graduate 34.3%	Breast
Djuric 2002 [36]	48	36–70 *	48/0	White 73%, African American 25%	College graduate 63%	Breast
Greenlee 2015 [37]	70	55.1 (9.1) vs. 58.0 (10.1)	70/0	White 41.2% vs. 38.9%, Black 20.6% vs. 30.6%, Mixed 14.7% vs. 16.7%, Native American 5.9% vs. 0.0%	College degree or higher 14.7% vs. 5.6%	Breast
Gruenigen 2012 [43]	75	57 (8.6) vs. 58.9 (10.9)	75/0	Caucasian 87.8% vs. 94.1%, African American 9.8% vs. 2.9%, Other 2.4% vs. 2.9%	College graduate or higher 39.0% vs. 41.2%	Gynaeco-logical
Harrigan 2016 [38]	100	59 (7.5)	100/0	Non-Hispanic White 91%	College degree 29%, graduate degree 37%	Breast
Hawkes 2013	410	64.9 (10.8) vs. 67.8 (9.2)	189/221	Not reported	Not reported	Colon
Kanera 2017 [48]	462	55.6 (11.5) vs. 56.2 (11.3)	369/93	Not reported	High education 34.2% vs. 27.7%	Mix
Kim 2011 [49]	45	44.6 (9.9) vs. 47.1 (7.3)	45/0	Not reported	Completed university 35.5%	Breast
Koutoukidis 2019 [40]	62	62.1 (8.3)	62/0	White 67% Asian 18% Black 8% Other 6%	Degree 47%	Gynaeco-logical
Morey 2009 [41]	641	73 (5.0) vs. 73.1 (5.1)	349/292	White 89.0% vs. 88.5%	Not reported	Mix °
Parekh 2018 [50]	59	58.5 (9.8) vs. 57 (10.8)	59/0	Asian 3.6% vs. 6.5%, Black or African American 28.6% vs. 16.1%, White 67.9% vs. 67.7, American Indian/Alaska Native 0.0% vs. 6.5%, Other race 0 vs. 3.2%	BA 25% vs. 35.5%, MA 39.3% vs. 35.5%, PhD 3.6%	Breast
Pierce 2007 [42]	3088	53.3 (9.8) vs. 53.0 (9.0)	3088/0	White 85% vs. 85.6%, African American 4% vs. 3.7%, Hispanic 5.7% vs. 5%, Asian American 3% vs. 3.2%, Mixed/other 2.3% vs. 2.5%	Not reported	Breast
Sheppard 2016 [51]	22	54.7 (9.8)	22/0	African American 100%	Not reported	Breast
Yun 2017 [52]	206	50.68 (9.4)	164/42	Korean 100%	College 48.53%	Mix °
Zick 2017 [53]	30	64.4 (10.0) vs. 10.4 (9.35)	30/0	White 93%	Not reported	Breast
Zuniga 2019 [50]	125	55.3 (10.3) vs. 58.4 (8.2)	125/0	Anglo 41.7% vs. 43.1% Latino 51.7% vs. 50.8% Other 6.7% vs. 6.2%	Some college/degree 40.0% vs. 26.2% college or higher 45.0% vs. 63.1%	Breast

Note: Intervention versus control was reported where the overall mean is not present; * range only report; ° breast, colon, prostate, and other cancer sites; ** calculation includes individual and group arm; *n*—total number of participants.

**Table 2:** Characteristics of the intervention.

Authors	Theory	Intervention Design and Resources	Behavioural Mediators Measured	Dietary Behaviour Beasures	Dietary Assessment Method	Physical Activity	Time Point (Months)
Blarigan 2020 [47]	SCT, TPB	Web based—text messages, printed materials, and personalised reports	Not reported	Vegetables, grains, fish, meat, sugary drinks, alcohol	24 h recall for 4 days	No	3, 6 follow up
Demark- Wahnefried 2006 [34]	TTM, SCT	Paper based—mailed information, telephone counselling, tailored workbook—information on current stage of diet/exercise, comparison to national guide and tips for change	Readiness to healthy diet, self-efficacy to healthy diet	Fruit and vegetables, BMI, DQI	3-day recall	Yes	6, 12 follow up
Demark- Wahnefried 2007 [33]	TTM, SCT	Paper based—mailed information, customized messages based on IT system programme reflecting TTM, SCT in tailored workbook, record logs, newsletter, advice for overcoming barriers, fun facts, graphic depiction of progress, update cards, pedometers	Self-efficacy of eating fruits and vegetables, stage of readiness to increase fruits and vegetables, behaviours practised at goal level	Fruit and vegetables, BMI, Fat (kcal), DQI	Diet history questionnaire	Yes	10
Demark- Wahnefried 2014 [35]	SCT, TTM	Mailed intervention—SCT tailored newsletters, messages on progress, reinforcement, encouragement, feedback, barriers, shoe chip, food records, activities logs, logbook, reference manual, website	Self-efficacy of adhering to healthy weight loss diet	EI, BMI, weight, WC, DQI	24 h recall	Yes	12
Djuric 2002 [36]	SCT	One to one/telephone counselling, group meetings, written resources but details not reported	Not reported	EI	3-day food diary	Yes	3, 12
Greenlee 2015 [37]	TTM, SCT	Group sessions on education, cooking and healthy shopping, resources—no details	Not reported	EI, fruit and vegetables, weight, BMI, WC, HC, WHP	24 h recall	No	3, 6 follow up
Gruenigen 2012 [43]	SCT	Group sessions, follow up newsletter, telephone, and emails to reinforce goals, resources—no detials	Not reported	fruit and vegetables, EI, weight, WC	24 h recall	Yes	6, 12 follow up
Harrigan 2016 [38]	SCT	One to one counselling, pedometer, scale, LEAN book—no details	Not reported	fruit and vegetables, weight, WC, % fat	FFQ	Yes	6, 12 follow up
Hawkes 2013 [39]	ACM, MIT	Telephone delivered health coaching sessions; postcard prompts; pedometer, book with educational information on lifestyle behaviours	Not reported	Fibre, fruit and vegetables, alcohol, BMI	FFQ	Yes	6, 12 follow up
Kanera 2017 [48]	SCT, TPB, SRT, IMC	Web-based programme with personalised feedback, online-evaluation session at the end	Not reported	Vegetable intake	Dutch standard questionnaire	Yes	12 follow up
Kim 2011 [49]	TTM	Telephone counselling, workbook on diet and exercise, heart rate monitor	Readiness to change	DQI	3-day recall	Yes	3
Koutoukidis 2018 [40]	SCT, CT	Group based intervention on eating pattern, balanced diet, portion size, food triggers, food labels and physical activity, study manual	Not reported	Healthy Eating Index	24 h recall	Yes	1.8, 5.5 follow up
Morey 2009 [41]	TTM, SCT	Paper based tailored workbook, newsletters, SCT telephone counselling, automated prompts, pedometer, exercise bands, table guide, record logs, workbook on diet and exercise	Not reported	Fruit and vegetables, weight, BMI	24 h recall	Yes	12
Parekh 2018 [50]	SCT	Group education sessions on diet, exercise and cooking classes, information brochures—no details	Not reported	Fruit and vegetables	Validated tool for fruit and vegetables	Yes	3
Pierce 2007 [42]	SCT	Telephone counselling sessions, cooking classes, newsletters—no details	Not reported	EI, fruit and vegetables, fibre, weight, adherence	24 h recall	No	72
Sheppard 2016 [51]	SCT, TPB, MIT	Group session—SCT, TBP, MIT telephone sessions, pedometers, notebook, resources—no details	Not reported	EI, fibre, weight, BMI, WC, HC, WHR	4-day food diary	Yes	3, 12
Yun 2017 [52]	TTM	Educational workshop, individual telephone coaching, partnership with cancer survivors, resources—no details	Not reported	Vegetable intake	Validated questionnaire	Yes	12
Zick 2017 [53]	SCT	Individualised telephone counselling, self-monitoring check list	Not reported	EI, vegetable intake, BMI	24 h recall	No	3
Zuniga 2019 [54]	TTM, MIT	Workshops with cooking demonstration, MIT telephone calls, newsletter, copies of lectures, TTM based sheet with goals	Not reported	EI, fibre, fruit and vegetables	Mediterranean diet questionnaire	No	6

Note: ACM—Acceptance Commitment Model, BMI—body mass index, DQI—Diet quality index, EI—energy intake, FFQ—food frequency questionnaire, HC—hip circumference, IMC—Integrated Model of Change, MIT—Motivational Interviewing, SCT—Social Cognitive Theory, SRT—Self-regulation theory, TTM-Trans-Theoretical Model of Change, TBP—Theory of Planned Behaviour, WC—waist circumference, WHR—waist o hip ratio.

**Table 3:** Behaviour change techniques taxonomy mapping of the studies.

BCT no.	BCTs/Authors	Blarigan 2020 [47]	Demark- Wahnefried 2006 [34]	Demark- Wahnefried 2007 [33]	Demark- Wahnefried 2014 [35]	Djuric 2002 [36]	Greenlee 2015 [37]	Gruenigen 2012 [43]	Harrigan 2016 [31]	Hawkes 2013 [39]	Kanera 2017 [48]	Kim 2011 [49]	Koutoukidis 2019 [40]	Morey 2009 [41]	Parekh 2018 [50]	Pierce 2007 [42]	Sheppard 2016 [51]	Yun 2017 [52]	Zick 2017 [53]	Zuniga 2019 [54]
1.1	Goal setting (behaviour)	1	1	1	1	-	-	-	-	-	-	-	1	-	-	-	-	-	-	1
1.2	Problem solving	-	-	1	1	1	1	-	-	1	1	1	-	1	-	1	1	-	-	-
1.3	Goal setting (outcomes)	-	1	-	-	1	-	1	1	1	1	1	-	1	-	1	1	1	1	-
1.4	Action planning	1	-	-	-	-	1	-	-	1	1	-	1	-	-	-	-	-	-	-
1.5	Review behaviour goal(s)	-	1	-	-	1	-	-	-	1	1	-	1	-	-	-	-	-	-	-
1.6	Discrepancy between current behaviour and goal	-	1	1	-	-	1	-	-	-	1	-	-	-	-	-	-	-	-	-
1.7	Review outcome goal(s)	-	1	-	-	-	-	-	-	-	-	-	-	-	-	1	-	-	-	-
1.8	Commitment	-	-	-	-	-	-	-	-	-	-	-	-	-	-	-	-	-	-	-
2.2	Feedback on behaviour	1	1	1	1	1	-	1		1	1	1	-	1	-	-	-	-	-	-
2.3	Self-monitoring of behaviour	-	-	1	1	1	-	-	1	1	1	1	1	1	-	1	1	-	1	-
2.7	Feedback on outcome(s) of behaviour	-	1	-	-	-	-	-	-	-	-	-	-	1	-	1	-	-	-	-
3.1	Social support (unspecified)	1	1	1	1	1	1	1	1	1	1	1	1	1	1	1	1	1	1	1
3.2	Social support (practical)	-	-	-	-	-	-	-	-	-	-	-	-	-	-	1	-	1	-	-
4.1	Instruction on how to perform the behaviour	1	1	1	1	1	1	1	1	1	1	1	1	1	1	1	1	1	-	1
5.1	Information on health consequences	-	-	-	-	-	1	-	-	1	1	1	1	-	1	-	-	-	-	1
5.4	Monitoring of emotional consequences	-	-	-	-	-	-	-	-	1	-	-	-	-	-	-	-	-	-	-
5.6	Information on emotional consequences	-	-	-	-	-	-	-	-	-	-	-	1	-	-	-	-	-	-	-
6.1	Demonstration of the behaviour	-	-	-	-	-	1	-	-	-	-	-	1	-	1	-	1	-	-	1
6.2	Social comparison	-	-	-	-	-	1	-	-	-	-	-	-	-	-	-	-	-	-	-
7.1	Prompts/cues	1	-	-	-	-	-	-	-	1	-	-	-	-	-	-	-	-	-	-
7.3	Reduce prompts/cues	-	-	-	-	1	-	-	-	-	-	-	-	-	-	-	-	-	-	-
8.1	Behavioural practice/rehearsal	-	-	-	-	-	-	-	-	-	-	-	1	-	1	1	-	-	-	-
8.7	Graded task	-	-	-	-	-	-	-	-	-	1	1	1	-	-	-	-	-	-	-
9.1	Credible source	1	1	1	1	1	1	1	1	1	1	1	1	1	1	1	1	1	1	1
9.2	Pros and cons	-	-	-	-	-	1	-	-	-	1	1	1	-	-	-	-	-	-	-
10.7	Self-incentive	-	-	-	-	-	-	-	-	-	-	1	1	-	-	-	-	-	-	-
10.9	Self-reward	-	-	-	-	-	-	-	-	-	-	1	1	-	-	-	-	-	-	-
11.2	Reduce negative emotions	-	-	-	-	-	-	-	-	1	-	-	-	-	-	-	-	-	-	-
12.3	Avoidance/reducing exposure to cues for the behaviour	-	-	-	-	1	-	-	-	-	-	1	1	-	-	-	-	-	-	-
13.2	Framing/reframing	-	-	-	-	-	-	-	-	-	-	-	1	-	-	-	-	-	-	-
	Total number	**7**	**10**	**8**	**7**	**10**	**10**	**5**	**5**	**13**	**13**	**13**	**17**	**8**	**6**	**10**	**6**	**5**	**4**	**6**

Note: BCT—Behaviour change techniques, (-) not used technique.

## Supplementary Materials

The following are available online at https://www.mdpi.com/2072-6643/13/2/612/s1. Table S1: Search Strategy; Table S2: GRADE PRO table for dietary outcomes; Table S3: GRADE PRO table for anthropometry outcomes; Figure S1: Mean energy intake; Figure S2: Mean fruit intake; Figure S3: Mean veg etable intake; Figure S4: Mean fruit and vegetable intake; Figure S5: Mean fibre intake; Figure S6: Diet Quality ndex; Figure S7: Mean weight; Figure S8: Mean Body Mass Index.
