# Supplementary material for: Systematic Review of Behaviour Change Theories Implementation in Dietary Interventions for People Who Have Survived Cancer
Source: Nutrients. 2021 Feb 13;13(2):612. doi: 10.3390/nu13020612 (PMC7917689; doi:10.3390/nu13020612)
Supplement: Supplementary file 1 [file nutrients-13-00612-s001.pdf]

Table S1. *CENTRAL* Search Strategy

#1	MeSH descriptor: [Neoplasms] explode all trees
#2	(cancer* or tumor* or tumour* or neoplas* or malignan* or carcinoma* or adenocarcinoma* or choriocarcinoma* or leukemia* or leukaemia* or metastat* or sarcoma* or teratoma*):ti,ab
#3	#1 or #2
#4	MeSH descriptor: [Diet] explode all trees
#5	MeSH descriptor: [Nutrition Assessment] explode all trees
#6	MeSH descriptor: [Nutrition Therapy] explode all trees
#7	MeSH descriptor: [Nutrition Disorders] explode all trees
#8	MeSH descriptor: [Food Habits] explode all trees
#9	MeSH descriptor: [Food Preferences] explode all trees
#10	MeSH descriptor: [Food] explode all trees
#11	(diet* or nutrition* or nutrient* or food* or feed* or eat* or drink*):ti,ab
#12	(fat* or carbohydrate* or protein* or fruit* or vegetable* or fibre* or fiber* or fish* or meat* or poultry or dairy or salt* or sugar* or cereal* or nut* or seed* or alcohol* or caGeine):ti
#13	(macrobiotic or ketogenic or vegetarian or (low adj (glycemic* or glycaemic*))):ti
#14	#5 or #6 or #7 or #8 or #9 or #10 or #11 or #12 or #13
#15	MeSH descriptor: [Survivors] explode all trees
#16	(survivor* or survival*):ti,ab
#17	#15 or #16
#18	#3 and #14 and #17
#19	Additional inclusion criterion on use of behaviour change theory was incorporated during the full text screening.

Table S2. GRADE PRO table for dietary outcomes.

**Outcomes**	**Anticipated Absolute Effects[*] (95% CI)**		**Number of Participants (Studies)**	**Certainty of the Evidence (GRADE)**
	**Risk with Usual Care**	**Risk with Dietary Changes**		
Energy intake (kcal) at 6 months	The mean energy intake (kcal) at 6 months was 1650.5 kcal	MD 104.72 kcal lower (246.35 lower to 36.91 higher)	3322 (4 RCTs)	⊕⊕⊕◯ MODERATE [a]
Energy intake (kcal) at 12 months	The mean energy intake (kcal) at 12 months was 1613 kcal	MD 109.69 kcal lower (251.09 lower to 31.7 higher)	3245 (4 RCTs)	⊕⊕⊕◯ MODERATE [a]
Fruit servings (per day) at 6 months	The mean fruit servings (per day) at 6 months was 2.65 serving	MD 0.62 serving higher (0.08 higher to 1.16 higher)	3157 (2 RCTs)	⊕⊕⊕◯ MODERATE [d]
Fruit servings (per day) at 12 months	The mean fruit servings (per day) at 12 months was 2.67 serving	MD 0.47 serving higher (0.13 lower to 1.07 higher)	3205 (3 RCTs)	⊕⊕⊕◯ MODERATE [d]
Vegetable servings (per day) at 6 months	The mean vegetable servings (per day) at 6 months was 2.3 serving	MD 2.7 grams higher (0.93 lower to 6.32 higher)	3200 (3 RCTs)	⊕⊕◯◯ LOW [e]

Outcomes	Anticipated Absolute Effects* (95% CI)		Number of Participants (Studies)	Certainty of the Evidence (GRADE)
	Risk with Usual Care	Risk with Dietary Changes		
Vegetable servings (per day) at 12 months	The mean vegetable servings (per day) at 12 months was 3.7 serving	MD 2.15 grams higher (1.38 lower to 5.67 higher)	3147 (2 RCTs)	⊕⊕◯◯ LOW [e]
Fruit and vegetable servings (per day) at 6 months	The mean fruit and vegetable servings (per day) at 6 months was 4.05 serving	MD 0.48 serving higher (0.06 lower to 1.02 higher)	237 (2 RCTs)	⊕⊕◯◯ LOW [b,f]
Fruit and vegetable servings (per day) at 12 months	The mean fruit and vegetable servings (per day) at 12 months was 5.0 serving	MD 0.38 serving higher (0.03 lower to 0.78 higher)	796 (4 RCTs)	⊕⊕⊕◯ MODERATE [g]
Fibre intake at 6 months	The mean fibre intake at 6 months was 16.65 grams	MD 3.56 grams higher (8.48 lower to 15.61 higher)	3213 (2 RCTs)	⊕⊕⊕◯ MODERATE [a]
Fibre intake at 12 months	The mean fibre intake at 12 months was 21.0 grams	MD 8 grams higher (7.3 higher to 8.7 higher)	3088 (1 RCT)	⊕⊕⊕◯ MODERATE [c]
Diet Quality Index at 12 months	The mean diet Quality Index at 12 months was 64.7 score	MD 3.62 score higher (1.95 higher to 5.30 higher)	719 (3 RCTs)	⊕⊕⊕◯ MODERATE [h]

*The risk in the intervention group (and its 95% confidence interval) is based on the assumed risk in the comparison group and the relative effect of the intervention (and its 95% CI). CI: Confidence interval; MD: Mean difference; SMD: Standardised mean difference. GRADE Working Group grades of evidence: High certainty: We are very confident that the true effect lies close to that of the estimate of the effect; Moderate certainty: We are moderately confident in the effect estimate: The true effect is likely to be close to the estimate of the effect, but there is a possibility that it is substantially different; Low certainty: Our confidence in the effect estimate is limited: The true effect may be substantially different from the estimate of the effect; Very low certainty: We have very little confidence in the effect estimate: The true effect is likely to be substantially different from the estimate of effect. Explanations: [a.] Downgraded due to wide variation in effect estimates across studies; [b.] Downgraded due to small study population; [c.] Downgraded one level as only one study is analysed; [d.] Downgraded one level due to inconsistency in studied population and design; [e.] Downgraded two levels due to large inconsistencies in studied population; [f.] Downgraded one level due to indirectness—primary aim of studies was not fruit and vegetable intake; [g.] Downgraded one level due to indirectness—primary aim of some studies was not fruit and vegetable intake; [h.] Downgraded one level due to risk of bias.

Table S3. GRADE PRO table for anthropometry outcomes.

Outcomes	Anticipated absolute effects* (95% CI)		№ of participants (studies)	Certainty of the evidence (GRADE)
	Risk with usual care	Risk with changes in anthropometry		
Weight (kg) at 12 months	The mean weight (kg) at 12 months was 82 kg	MD 0.89 kg lower (2.12 lower to 0.34 higher)	3245 (4 RCTs)	⊕⊕⊕◯ MODERATE [a]
Body mass index (kg/m^2) at 12 months	The mean body mass index (kg/m^2) at 12 months was 29.6 kg/m2	MD 0.79 kg/m2 lower (1.5 lower to 0.07 lower)	777 (4 RCTs)	⊕⊕⊕◯ MODERATE [b]
Waist circumference (cm) at 6 months	The mean waist circumference (cm) at 6 months was 98.5 cm	MD 0.33 cm lower (4.79 lower to 4.14 higher)	109 (2 RCTs)	⊕⊕◯◯ LOW [a,c]

Outcomes	Anticipated absolute effects* (95% CI)		№ of participants (studies)	Certainty of the evidence (GRADE)
	Risk with usual care	Risk with changes in anthropometry		
Mean waist circumference (cm) - 12 months	The mean waist circumference (cm) - Waist ratio at 12 months was 99.2 cm	MD 3.47 cm lower (7.61 lower to 0.67 higher)	148 (3 RCTs)	⊕⊕◯◯ LOW [a,c]

*The risk in the intervention group (and its 95% confidence interval) is based on the assumed risk in the comparison group and the relative effect of the intervention (and its 95% CI). CI: Confidence interval; MD: Mean difference. GRADE Working Group grades of evidence: High certainty: We are very confident that the true effect lies close to that of the estimate of the effect; Moderate certainty: We are moderately confident in the effect estimate: The true effect is likely to be close to the estimate of the effect, but there is a possibility that it is substantially different; Low certainty: Our confidence in the effect estimate is limited: The true effect may be substantially different from the estimate of the effect; Very low certainty: We have very little confidence in the effect estimate: The true effect is likely to be substantially different from the estimate of effect. Explanations: [a.] Downgraded one level due to differences in studied population. [b.] Downgraded one level due to risk of bias assessment. [c.] Downgraded one level due to small sample size.

Figure S1. Mean energy intake (kcal) at 6 and 12 months.

Study or Subgroup	Dietary intervention Mean	SD	Total	Usual care Mean	SD	Total	Weight	Mean Difference IV, Random, 95% CI
**1.1.1 Energy intake at 6 mo**								
Demark-Wahnefried 2014	1,195	365	23	1,337	452	17	18.0%	-142.00 [-403.57, 119.57]
Gruenigen 2012	1,635	579	41	1,845	625	28	15.7%	-210.00 [-501.55, 81.55]
Pierce 2007	1,619	549	1537	1,615	591	1551	45.1%	4.00 [-36.23, 44.23]
Zuniga 2019	1,578.3	663.1	60	1,805.3	636.9	65	21.1%	-227.00 [-455.31, 1.31]
**Subtotal (95% CI)**			**1661**			**1661**	**100.0%**	**-104.72 [-246.35, 36.91]**
Heterogeneity: Tau² = 11145.61; Chi² = 6.72, df = 3 (P = 0.08); I² = 55%								
Test for overall effect: Z = 1.45 (P = 0.15)								
**1.1.2 Energy intake at 12 mo**								
Demark-Wahnefried 2014	1,189	429	23	1,400	487	17	15.8%	-211.00 [-501.40, 79.40]
Greenlee 2015	1,440.5	440.2	29	1,640.4	390.9	29	22.7%	-199.90 [-414.16, 14.36]
Gruenigen 2012	1,607	496	35	1,807	632	24	15.0%	-200.00 [-501.55, 101.55]
Pierce 2007	1,603	392	1537	1,605	433	1551	46.5%	-2.00 [-31.13, 27.13]
**Subtotal (95% CI)**			**1624**			**1621**	**100.0%**	**-109.69 [-251.09, 31.70]**
Heterogeneity: Tau² = 10977.29; Chi² = 6.67, df = 3 (P = 0.08); I² = 55%								
Test for overall effect: Z = 1.52 (P = 0.13)								

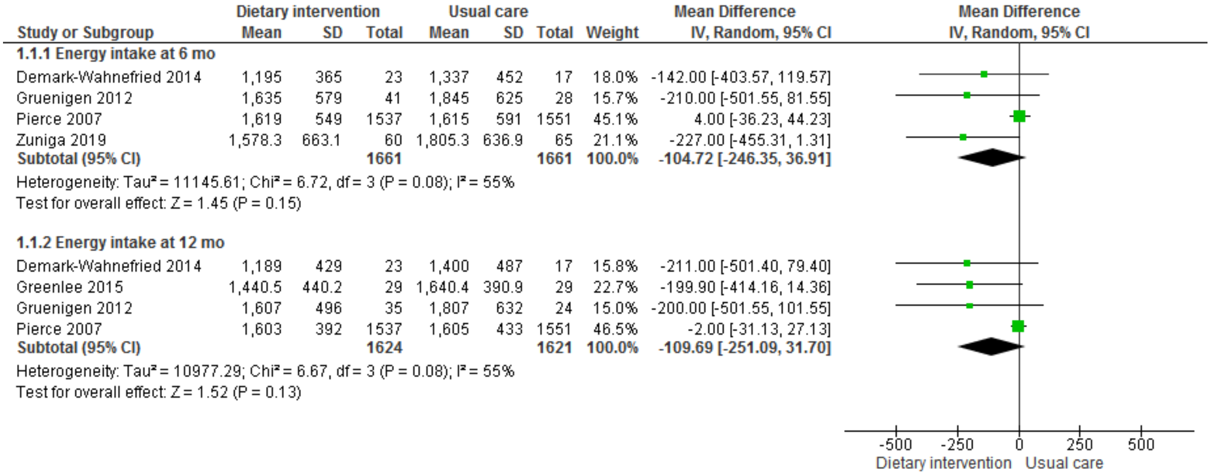

Notes: SD=standard deviation; IV=weighted mean difference; CI=confidence interval; df=degrees of freedom; Chi2=chi-square statistic; p=p value; I2=I-square heterogeneity statistic; Z=Z statistic

Figure S2. Mean fruit intake (servings) at 6 and 12 months.

Study or Subgroup	Dietary intervention Mean	SD	Total	Usual care Mean	SD	Total	Weight	Mean Difference IV, Random, 95% CI
**1.2.1 Fruit servings (per day) at 6 months**								
Gruenigen 2012	1.9	1.6	41	1.7	1.6	28	30.4%	0.20 [-0.57, 0.97]
Pierce 2007	4.4	3.1	1537	3.6	3.2	1551	69.6%	0.80 [0.58, 1.02]
**Subtotal (95% CI)**			**1578**			**1579**	**100.0%**	**0.62 [0.08, 1.16]**
Heterogeneity: Tau² = 0.10; Chi² = 2.16, df = 1 (P = 0.14); I² = 54%								
Test for overall effect: Z = 2.24 (P = 0.03)								
**1.2.2 Fruit servings (per day) at 12 months**								
Greenlee 2015	2.7	1.5	29	2.8	1.9	29	25.6%	-0.10 [-0.98, 0.78]
Gruenigen 2012	2.1	2.5	35	1.8	1.8	24	19.6%	0.30 [-0.80, 1.40]
Pierce 2007	4.2	2.4	1537	3.4	2	1551	54.8%	0.80 [0.64, 0.96]
**Subtotal (95% CI)**			**1601**			**1604**	**100.0%**	**0.47 [-0.13, 1.07]**
Heterogeneity: Tau² = 0.17; Chi² = 4.59, df = 2 (P = 0.10); I² = 56%								
Test for overall effect: Z = 1.54 (P = 0.12)								

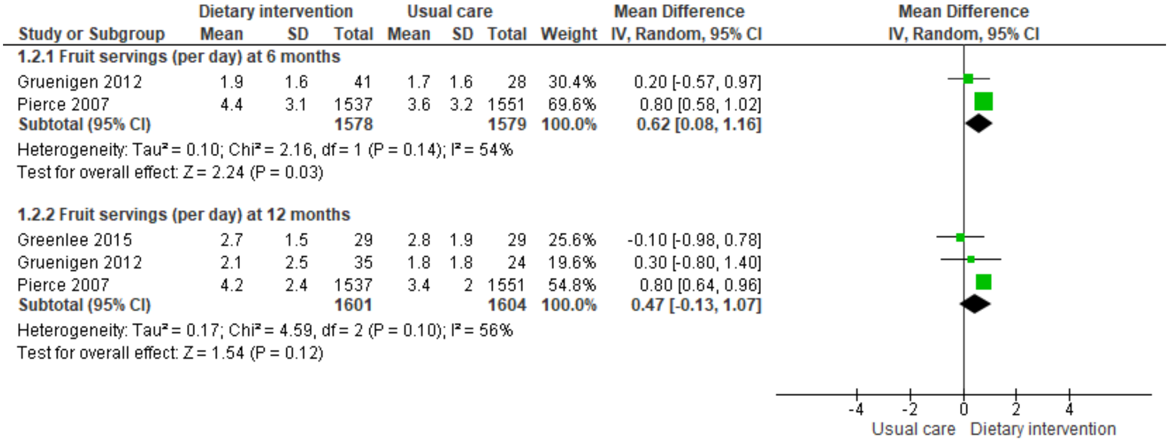

Notes: SD=standard deviation; IV=weighted mean difference; CI=confidence interval; df=degrees of freedom; Chi2=chi-square statistic; p=p value; I2=I-square heterogeneity statistic; Z=Z statistic

Figure S3. Mean vegetable intake (servings) at 6 and 12 months.

Study or Subgroup	Dietary intervention Mean	SD	Total	Usual care Mean	SD	Total	Weight	Mean Difference IV, Random, 95% CI
**1.3.1 Vegetable servings (per day) at 6 months**								
Blarigan 2020	0.3	0	22	-0.2	0	22		Not estimable
Gruenigen 2012	4	2.5	41	3.2	2.4	28	48.7%	0.80 [-0.37, 1.97]
Pierce 2007	8.4	0.13	1537	3.9	0.07	1551	51.3%	4.50 [4.49, 4.51]
**Subtotal (95% CI)**			**1600**			**1601**	**100.0%**	**2.70 [-0.93, 6.32]**
Heterogeneity: Tau² = 6.67; Chi² = 38.22, df = 1 (P < 0.00001); I² = 97%								
Test for overall effect: Z = 1.46 (P = 0.14)								
**1.3.2 Vegetable servings (per day) at 12 months**								
Gruenigen 2012	3.7	2.5	35	3.4	2	24	48.7%	0.30 [-0.85, 1.45]
Pierce 2007	7.8	0.09	1537	3.9	0.05	1551	51.3%	3.90 [3.89, 3.91]
**Subtotal (95% CI)**			**1572**			**1575**	**100.0%**	**2.15 [-1.38, 5.67]**
Heterogeneity: Tau² = 6.31; Chi² = 37.54, df = 1 (P < 0.00001); I² = 97%								
Test for overall effect: Z = 1.19 (P = 0.23)								

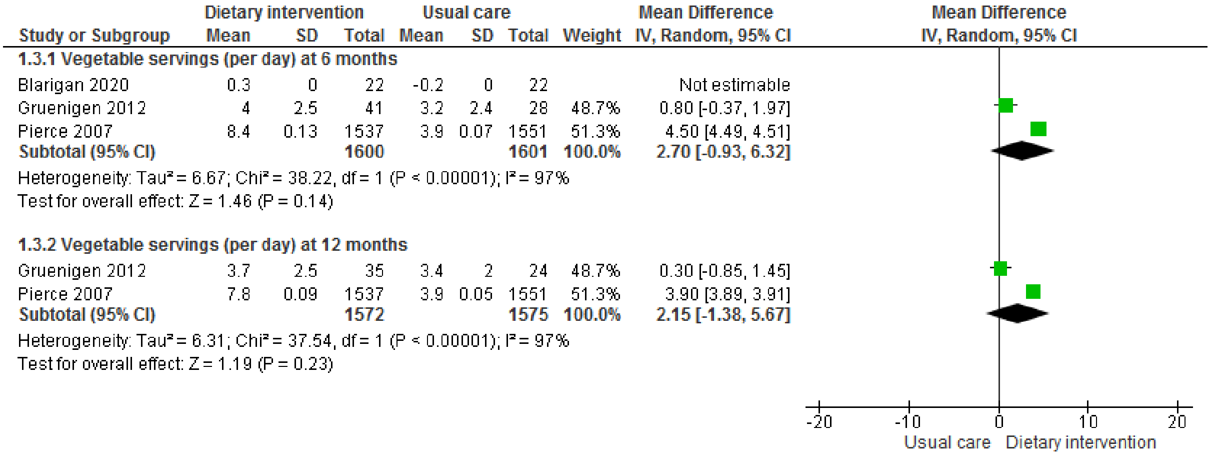

Notes: SD=standard deviation; IV=weighted mean difference; CI=confidence interval; df=degrees of freedom; Chi2=chi-Scheme 2. I-square heterogeneity statistic; Z=Z statistic.

Figure S4. Mean fruit and vegetable intake (servings) at 6 and 12 months.

Study or Subgroup	Dietary intervention Mean	SD	Total	Usual care Mean	SD	Total	Weight	Mean Difference IV, Random, 95% CI
**1.4.1 Fruit and vegetable servings (per day) at 6 months**								
Demark-Wahnefried 2006	3.6	2	82	3.2	1.8	86	86.9%	0.40 [-0.18, 0.98]
Gruenigen 2012	5.9	3.2	41	4.9	3	28	13.1%	1.00 [-0.48, 2.48]
**Subtotal (95% CI)**			**123**			**114**	**100.0%**	**0.48 [-0.06, 1.02]**
Heterogeneity: Tau² = 0.00; Chi² = 0.55, df = 1 (P = 0.46); I² = 0%								
Test for overall effect: Z = 1.75 (P = 0.08)								
**1.4.2 Fruit and vegetable servings (per day) at 12 months**								
Demark-Wahnefried 2006	3.5	2.1	77	3.6	2.1	83	31.0%	-0.10 [-0.75, 0.55]
Demark-Wahnefried 2007	6.1	2.8	253	5.6	2.6	266	50.8%	0.50 [0.03, 0.97]
Greenlee 2015	6.8	2.2	29	5.8	2.3	29	11.4%	1.00 [-0.16, 2.16]
Gruenigen 2012	5.6	3.6	35	5	2.4	24	6.8%	0.60 [-0.93, 2.13]
**Subtotal (95% CI)**			**394**			**402**	**100.0%**	**0.38 [-0.03, 0.78]**
Heterogeneity: Tau² = 0.03; Chi² = 3.52, df = 3 (P = 0.32); I² = 15%								
Test for overall effect: Z = 1.82 (P = 0.07)								

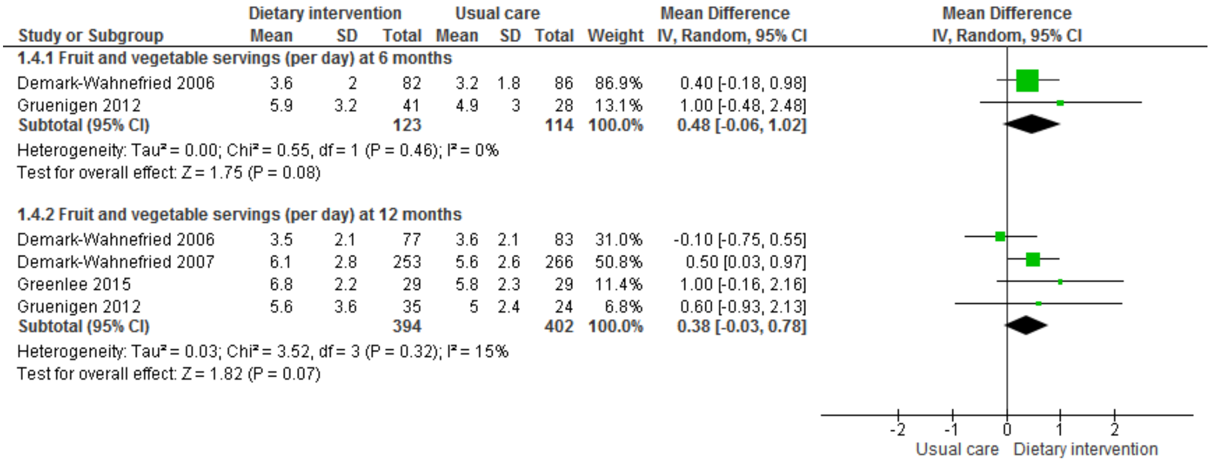

Notes: SD=standard deviation; IV=weighted mean difference; CI=confidence interval; df=degrees of freedom; Chi2=chi-square statistic; p=p value; I2=I-square heterogeneity statistic; Z=Z statistic

Figure S5. Mean fibre intake (grams) at 6 and 12 months.

Study or Subgroup	Dietary intervention Mean	SD	Total	Usual care Mean	SD	Total	Weight	Mean Difference IV, Random, 95% CI
**1.5.1 Fibre intake at 6 months**								
Pierce 2007	30.9	15.6818	1537	21.4	11.8148	1551	51.7%	9.50 [8.52, 10.48]
Zuniga 2019	9.1	13.2	60	11.9	12.9	65	48.3%	-2.80 [-7.38, 1.78]
**Subtotal (95% CI)**			**1597**			**1616**	**100.0%**	**3.56 [-8.48, 15.61]**
Heterogeneity: Tau² = 72.79; Chi² = 26.48, df = 1 (P < 0.00001); I² = 96%								
Test for overall effect: Z = 0.58 (P = 0.56)								
**1.5.2 Fibre intake at 12 months**								
Pierce 2007	29	10.9773	1537	21	8.6642	1551	100.0%	8.00 [7.30, 8.70]
**Subtotal (95% CI)**			**1537**			**1551**	**100.0%**	**8.00 [7.30, 8.70]**
Heterogeneity: Not applicable								
Test for overall effect: Z = 22.47 (P < 0.00001)								

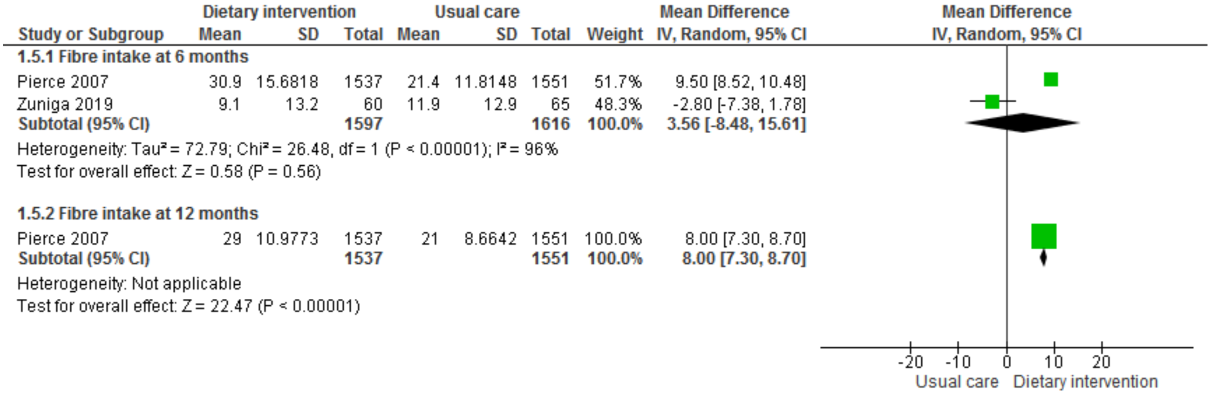

Notes: SD=standard deviation; IV=weighted mean difference; CI=confidence interval; df=degrees of freedom; Chi2=chi-square statistic; p=p value; I2=I-square heterogeneity statistic; Z=Z statistic

Figure S6. Diet Quality Index at 6 and 12 months.

Study or Subgroup	Dietary intervention Mean	SD	Total	Usual care Mean	SD	Total	Weight	Mean Difference IV, Random, 95% CI
**1.6.1 Diet Quality Index at 6 months**								
Demark-Wahnefried 2006	69.8	13.9	82	64.6	14.7	86	100.0%	5.20 [0.88, 9.52]
**Subtotal (95% CI)**			**82**			**86**	**100.0%**	**5.20 [0.88, 9.52]**
Heterogeneity: Not applicable								
Test for overall effect: Z = 2.36 (P = 0.02)								
**1.6.2 Diet Quality Index at 12 months**								
Demark-Wahnefried 2006	67.5	13.6	77	66.6	13.1	83	16.2%	0.90 [-3.24, 5.04]
Demark-Wahnefried 2007	72.8	10.6	253	68.7	10.9	266	78.2%	4.10 [2.25, 5.95]
Demark-Wahnefried 2014	63.7	11.9	23	58.9	10.7	17	5.6%	4.80 [-2.24, 11.84]
**Subtotal (95% CI)**			**353**			**366**	**100.0%**	**3.62 [1.95, 5.30]**
Heterogeneity: Tau² = 0.04; Chi² = 2.02, df = 2 (P = 0.36); I² = 1%								
Test for overall effect: Z = 4.24 (P < 0.0001)								

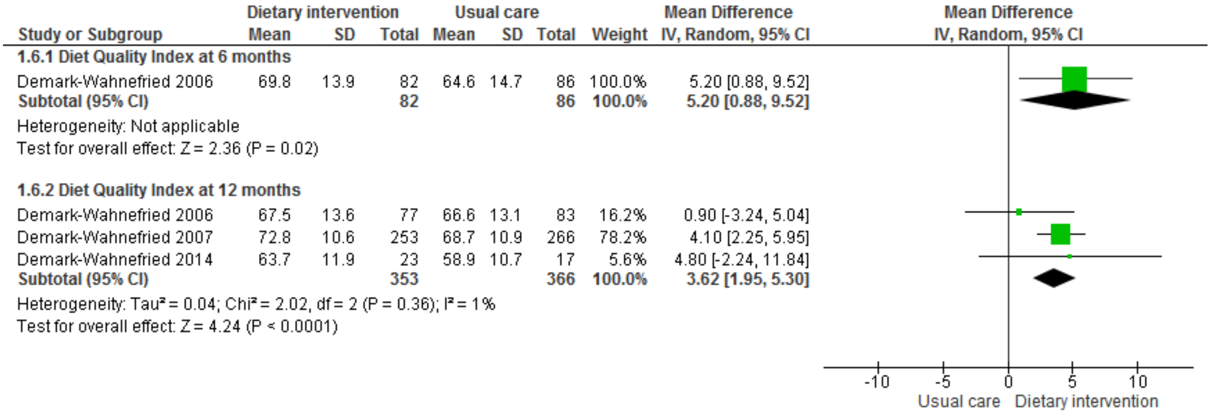

Notes: SD=standard deviation; IV=weighted mean difference; CI=confidence interval; df=degrees of freedom; Chi2=chi-square statistic; p=p value; I2=I-square heterogeneity statistic; Z=Z statistic

Figure S7. Mean weight (kg) at 6 and 12 months.

	Dietary intervention			Usual care				Mean Difference
**Study or Subgroup**	**Mean**	**SD**	**Total**	**Mean**	**SD**	**Total**	**Weight**	**IV, Random, 95% CI**
**2.1.2 Weight at 6 months**								
Gruenigen 2012	91.8	19.3	41	94.6	23.8	28	100.0%	-2.80 [-13.41, 7.81]
**Subtotal (95% CI)**			**41**			**28**	**100.0%**	**-2.80 [-13.41, 7.81]**
Heterogeneity: Not applicable								
Test for overall effect: Z = 0.52 (P = 0.61)								
**2.1.3 Weight at 12 months**								
Demark-Wahnefried 2014	79.7	10.2	23	80.7	10.1	17	3.8%	-1.00 [-7.36, 5.36]
Greenlee 2015	74.2	17.8	29	78.1	15.9	29	2.0%	-3.90 [-12.59, 4.79]
Gruenigen 2012	92.7	20.1	35	95.4	25.4	24	1.0%	-2.70 [-14.85, 9.45]
Pierce 2007	73	17.7	1537	73.8	18.5	1551	93.2%	-0.80 [-2.08, 0.48]
**Subtotal (95% CI)**			**1624**			**1621**	**100.0%**	**-0.89 [-2.12, 0.34]**
Heterogeneity: Tau² = 0.00; Chi² = 0.57, df = 3 (P = 0.90); I² = 0%								
Test for overall effect: Z = 1.41 (P = 0.16)								

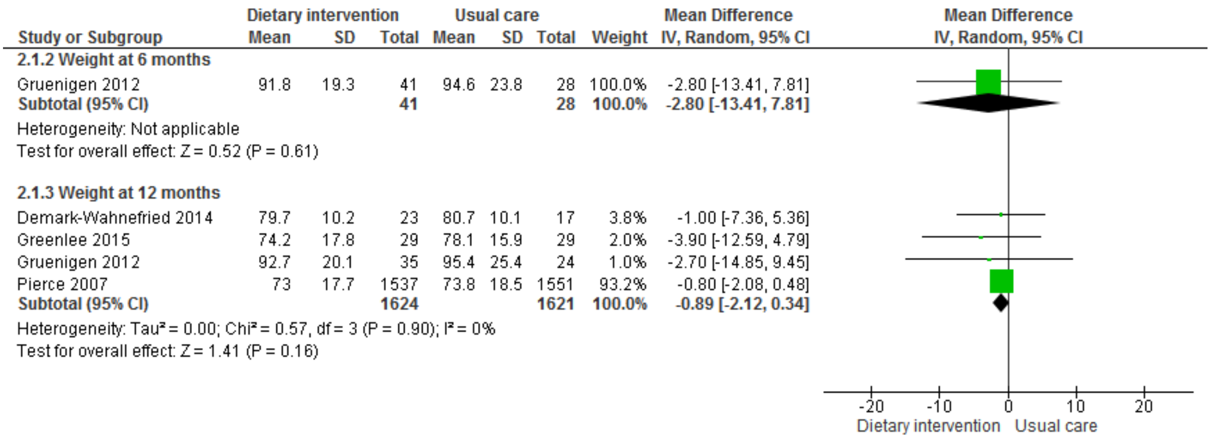

Notes: SD=standard deviation; IV=weighted mean difference; CI=confidence interval; df=degrees of freedom; Chi2=chi-square statistic; p=p value; I2=I-square heterogeneity statistic; Z=Z statistic

Figure S8. Mean Body Mass Index (kg/m²) at 6 and 12 months.

	Dietary intervention			Usual care				Mean Difference
**Study or Subgroup**	**Mean**	**SD**	**Total**	**Mean**	**SD**	**Total**	**Weight**	**IV, Random, 95% CI**
**2.2.2 Body mass index at 6 months**								
Demark-Wahnefried 2006	27.6	5.3	82	28.3	5	86	100.0%	-0.70 [-2.26, 0.86]
**Subtotal (95% CI)**			**82**			**86**	**100.0%**	**-0.70 [-2.26, 0.86]**
Heterogeneity: Not applicable								
Test for overall effect: Z = 0.88 (P = 0.38)								
**2.2.3 Body mass index at 12 months**								
Demark-Wahnefried 2006	27.6	5.2	77	28.7	5.2	83	19.6%	-1.10 [-2.71, 0.51]
Demark-Wahnefried 2007	27.1	5	253	27.8	5.3	266	64.9%	-0.70 [-1.59, 0.19]
Demark-Wahnefried 2014	30.1	4	23	30.4	3.1	17	10.5%	-0.30 [-2.50, 1.90]
Greenlee 2015	29.9	6.6	29	31.6	5.8	29	5.0%	-1.70 [-4.90, 1.50]
**Subtotal (95% CI)**			**382**			**395**	**100.0%**	**-0.79 [-1.50, -0.07]**
Heterogeneity: Tau² = 0.00; Chi² = 0.68, df = 3 (P = 0.88); I² = 0%								
Test for overall effect: Z = 2.16 (P = 0.03)								

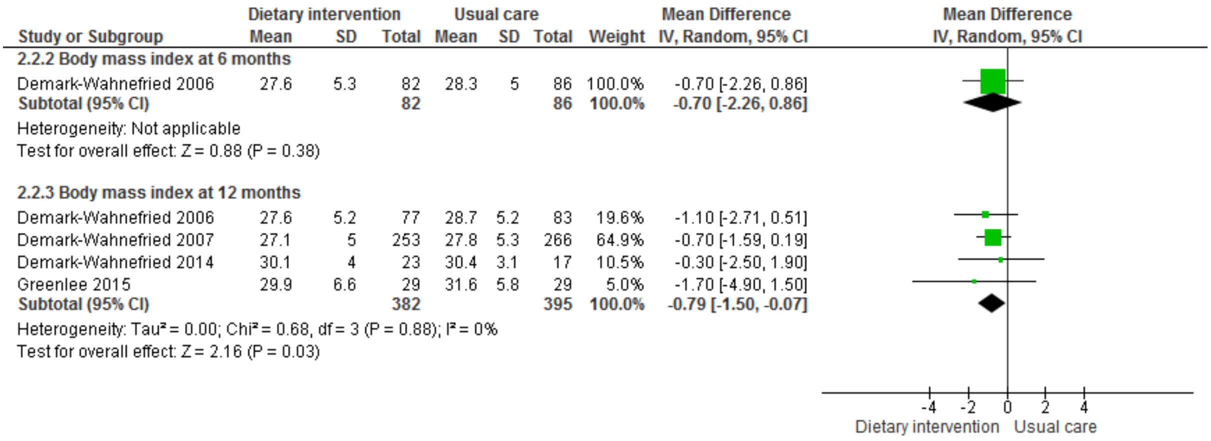

Notes: SD=standard deviation; IV=weighted mean difference; CI=confidence interval; df=degrees of freedom; Chi2=chi-square statistic; p=p value; I2=I-square heterogeneity statistic; Z=Z statistic

Figure S9. Mean waist circumference (cm) at 6 and 12 months.

	Dietary intervention			Usual care				Mean Difference
**Study or Subgroup**	**Mean**	**SD**	**Total**	**Mean**	**SD**	**Total**	**Weight**	**IV, Random, 95% CI**
**2.3.2 Waist ratio at 6 months**								
Demark-Wahnefried 2014	92.6	8.7	23	93.8	9.1	17	60.3%	-1.20 [-6.80, 4.40]
Gruenigen 2012	104.4	12.5	41	103.4	15.5	28	39.7%	1.00 [-5.90, 7.90]
**Subtotal (95% CI)**			**64**			**45**	**100.0%**	**-0.33 [-4.67, 4.02]**
Heterogeneity: Tau² = 0.00; Chi² = 0.24, df = 1 (P = 0.63); I² = 0%								
Test for overall effect: Z = 0.15 (P = 0.88)								
**2.3.3 Waist ratio at 12 months**								
Demark-Wahnefried 2014	90.7	7.4	23	93.7	9.7	17	46.0%	-3.00 [-8.51, 2.51]
Greenlee 2015	92.8	12.4	23	100.3	13	26	30.1%	-7.50 [-14.62, -0.38]
Gruenigen 2012	104.4	15	35	103.6	16	24	23.9%	0.80 [-7.30, 8.90]
**Subtotal (95% CI)**			**81**			**67**	**100.0%**	**-3.44 [-7.63, 0.74]**
Heterogeneity: Tau² = 1.99; Chi² = 2.33, df = 2 (P = 0.31); I² = 14%								
Test for overall effect: Z = 1.61 (P = 0.11)								

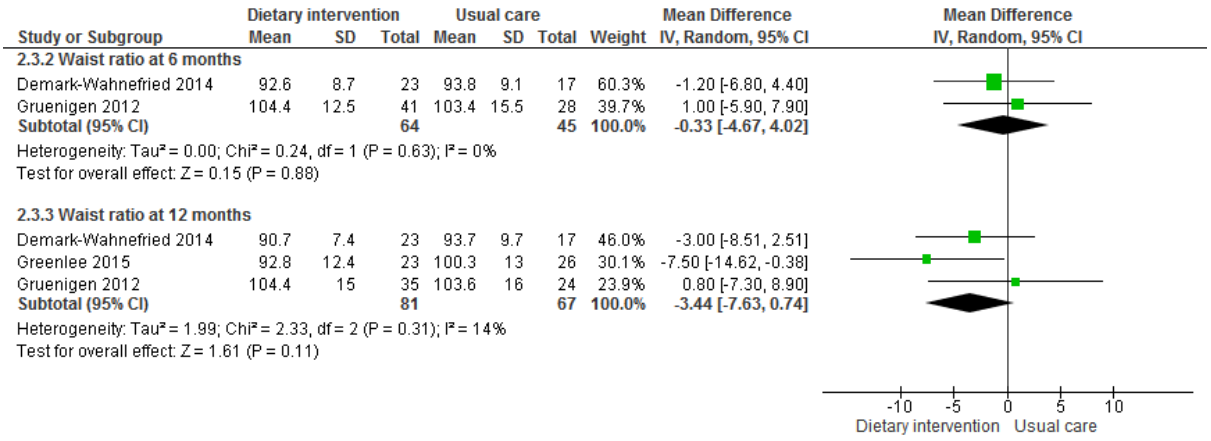

Notes: SD=standard deviation; IV=weighted mean difference; CI=confidence interval; df=degrees of freedom; Chi2=chi-square statistic; p=p value; I2=I-square heterogeneity statistic; Z=Z statistic
